# P-1776. Combatting the brain-eating ameba Balamuthia mandrillaris: from bench to bedside to public health

**DOI:** 10.1093/ofid/ofaf695.1946

**Published:** 2026-01-11

**Authors:** Natasha Spottiswoode, Kaitlin Marquis, Angela Detweiler, Norma Neff, Samuel Lord, Joseph DeRisi

**Affiliations:** University of California, San Francisco, San Francisco, CA; Chan Zuckerberg Biohub, San Francisco, CA, USA., San Francisco, California; Insitro, San Francisco, CA, USA., San Francisco, California; Chan Zuckerberg Biohub, San Francisco, CA, USA., San Francisco, California; Department of Cellular and Molecular Pharmacology, Howard Hughes Medical Institute, University of California, San Francisco, CA, USA., San Francisco, California; Department of Biochemistry and Biophysics, University of California, San Francisco, CA, USA, San Francisco, California

## Abstract

**Background:**

*B. mandrillaris* is a free-living ameba that causes granulomatous amebic encephalitis (GAE), an infectious syndrome with a mortality rate of >90%. Treatment for GAE has been hampered by limited understanding of the molecular mechanisms of the pathogen and a lack of effective therapeutics. Recently, the quinolone nitroxoline (NTX) was identified in a drug screen as effective against *B. mandrillaris,* and successfully used to treat two US patients.

**Figure 1.**
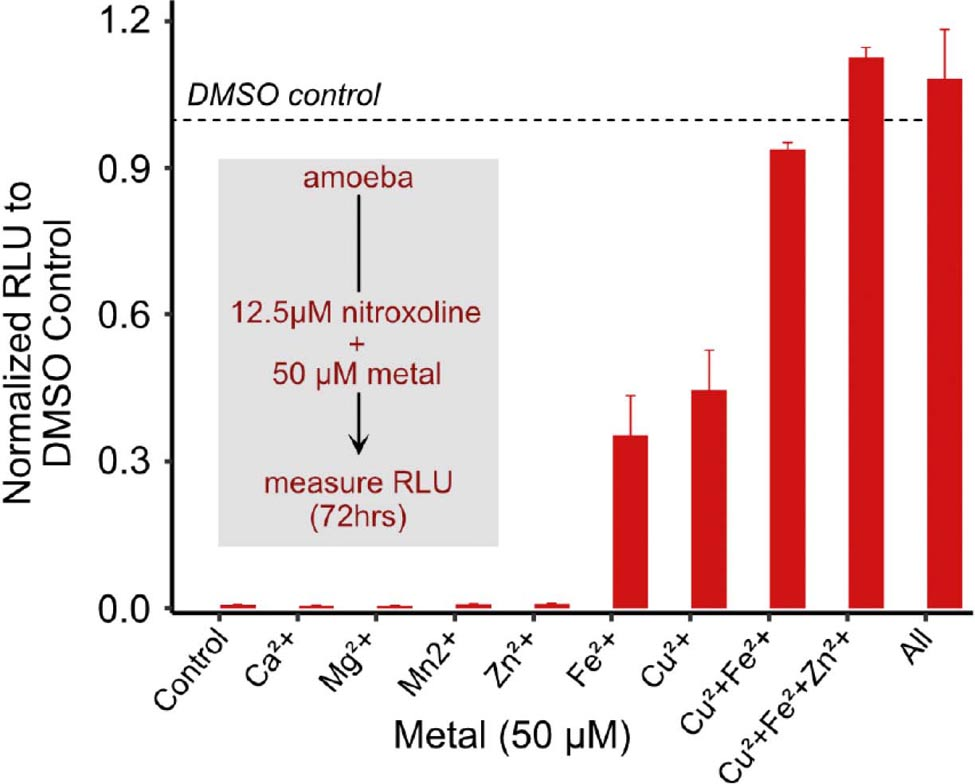
Metal supplementation rescues nitroxoline mediated inhibition in B. mandrillaris. Exogenous metal supplementation rescues nitroxoline mediated inhibition. Exogenous metals were added to nitroxoline or DMSO treated trophozoites and viability was assessed 72 hours later using a luciferase-based viability assay. Relative light units (RLU) are normalized to respective DMSO vehicle controls (n = 3).

**Figure 2.**
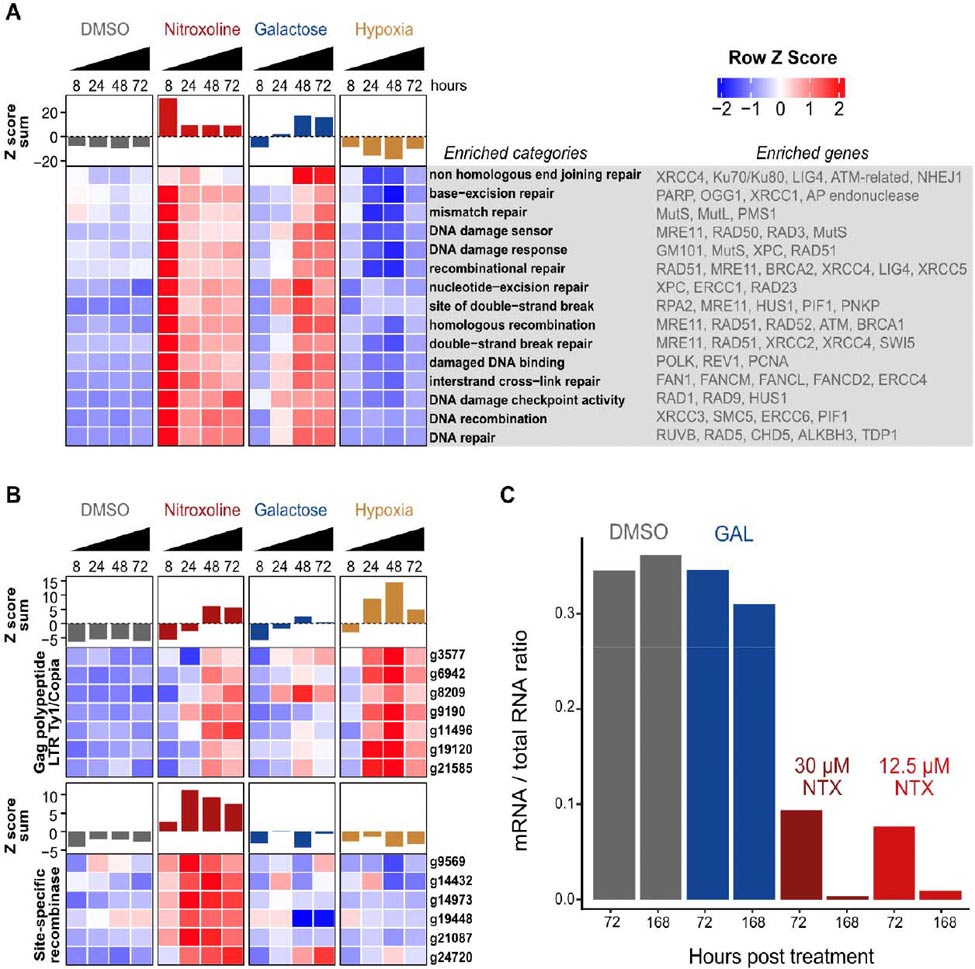
Nitroxoline induces genomic stress and perturbs global mRNA transcription. (A) Nitroxoline induces early and excessive expression of DNA damage and repair genes at 8 hours post treatment. Expression patterns for 484 unique DNA damage and repair associated genes were collapsed into enriched gene categories using high confidence annotations. Each category contains at least 10 unique genes and all row Z scores were summed to provide overall expression profiles. (B) Nitroxoline and hypoxia upregulate transcription from select transposons. Functional transposons were identified using EDTA clustered into categories sharing 70% sequence identity and manually annotated. Row Z scores for each gene were summed to provide overall expression profiles of the clusters. (C) Global mRNA transcription is impaired in nitroxoline induced encystment. Encystment was stimulated with 12% galactose (GAL), 12.5 uM nitroxoline (NTX) or 30 uM nitroxoline (NTX) for 72 and 168 hours and compared to DMSO treated trophozoites. Total RNA was extracted and the proportion of short lived mRNA reads were normalized to total RNA reads for each sample.

**Methods:**

We used transcriptomics, growth assays, chemical complementation, cyst integrity assessments, and scanning electron microscopy to assess effects of NTX on *B. mandrillaris.* To support this work, we created a new draft *B. mandrillaris* genome, available at NCBI BioProject PRJNA1206197.

**Figure 3.**
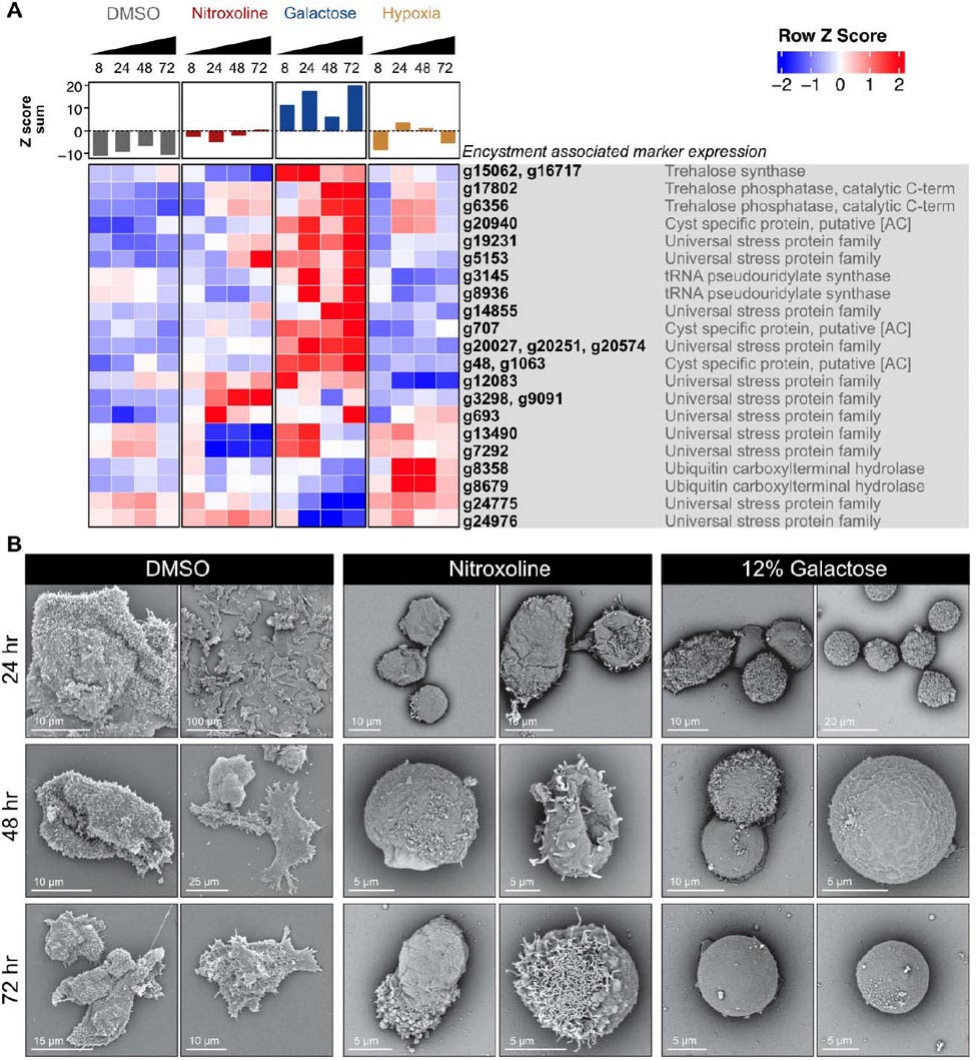
Nitroxoline perturbs the encystment process and undermines cyst structural integrity. (A) Nitroxoline fails to upregulate several encystment associated marker genes. Trehalose synthesis and universal stress protein family genes associated with amebic encystment were identified from the list of differentially expressed genes (Figure 1) and visualized in a heatmap. Identical gene copies (more than 99% identity) were averaged before computing row Z scores. Row Z scores for each unique gene were summed to provide an overall summary of the expression pattern. (B) Nitroxoline induced cysts show altered morphologies compared to galactose induced cysts. Balamuthia trophozoites were treated with 12.5 uM nitroxoline, 12% galactose, or DMSO vehicle control and samples were fixed for scanning electron microscopy at 24, 48, and 72 hours post treatment. Cells were imaged using a Phenom Pharos G2 desktop scanning electron microscope.

**Results:**

Nitroxoline induced killing of *B. mandrillaris* was rescued by supplementation with copper and/or iron (Fig. 1). Nitroxoline treatment is associated with increased expression of DNA damage and repair genes (Fig. 2A), increased transposon transcription (Fig. 2B) and depressed global mRNA transcription (Fig. 2C), all consistent with parasite death. *B. mandrillaris* forms a tough, resistant cyst form under stress, but key encystment genes failed to upregulate in NTX-treated *B. mandrillaris* (Fig. 3A), and electron microscopy demonstrated disrupted cyst morphology in NTX-treated *B. mandrillaris* (Fig 3B).

**Conclusion:**

NTX sequesters iron and copper ions from *B. mandrillaris*, causing metabolic and genomic disarray, disrupting formation of the protective cyst, and ultimately leading to parasite death. These data contribute to a rational basis for including NTX in *B. mandrillaris* treatment regimens. Nitroxoline is now available for patients in the United States through an expanded-access Investigational New Drug protocol held by the Centers for Disease Control and Prevention.

**Disclosures:**

All Authors: No reported disclosures

